# Metabolomics identifies increases in the acylcarnitine profiles in the plasma of overweight subjects in response to mild weight loss: a randomized, controlled design study

**DOI:** 10.1186/s12944-018-0887-1

**Published:** 2018-10-15

**Authors:** Miso Kang, Hye Jin Yoo, Minjoo Kim, Minkyung Kim, Jong Ho Lee

**Affiliations:** 1 National Leading Research Laboratory of Clinical Nutrigenetics/Nutrigenomics, Department of Food and Nutrition, College of Human Ecology, Yonsei University, 50 Yonsei-ro, Seodaemun-gu, Seoul, 03722 South Korea; 2 Department of Food and Nutrition, Brain Korea 21 PLUS Project, College of Human Ecology, Yonsei University, Seoul, 03722 South Korea; 3 Research Center for Silver Science, Institute of Symbiotic Life-TECH, Yonsei University, Seoul, 03722 South Korea

**Keywords:** Low calorie diet, Metabolomics, Mild weight loss, Visceral fat area, Acylcarnitine

## Abstract

**Background:**

Using metabolomics technique to analyze the response to a dietary intervention generates valuable information concerning the effects of the prescribed diet on metabolic regulation. To determine whether low calorie diet (LCD)-induced weight reduction causes changes in plasma metabolites and metabolic characteristics.

**Methods:**

Overweight subjects consumed a LCD (*n* = 47) or a weight maintenance diet (control, *n* = 50) in a randomized, controlled design study with a 12-week clinical intervention period. Plasma samples were analyzed using an UPLC-LTQ-Orbitrap MS.

**Results:**

The 12-week LCD intervention resulted in significant mild weight loss, with an 8.3% and 10.6% reduction observed in the visceral fat area (VFA) at the level of the lumbar vertebrae L1 and L4, respectively. The LCD group showed a significant increase in the mean change of serum free fatty acids compared to the control group. In the LCD group, we observed a significant increase in the acylcarnitine (AC) levels, including hexanoylcarnitine, L-octanoylcarnitine, 9-decenoylcarnitine, trans-2-dodecenoylcanitine, dodecanoylcarnitine, 3,5-tetradecadiencarnitine, cis-5-tetradecenoylcarnitine, 9,12-hexadecadienoylcarnitine, and 9-hexadecenoylcarnitne at the 12-week follow-up assessment. When the plasma metabolite changes from baseline were compared between the control and LCD groups, the LCD group showed significant increases in hexanoylcarnitine, L-octanoylcarnitine, trans-2-dodecenoylcanitine, and 3,5-tetradecadiencarnitine than the control group. Additionally, the changes in these ACs in the LCD group strongly negatively correlated with the changes in the VFA at L1 and/or L4.

**Conclusion:**

Mild weight loss from 12-week calorie restriction increased the plasma levels of medium- and long-chain ACs. These changes were coupled with a decrease in VFA and an increase in free fatty acids.

**Trial registration:**

NCT03135132; April 26, 2017.

**Electronic supplementary material:**

The online version of this article (10.1186/s12944-018-0887-1) contains supplementary material, which is available to authorized users.

## Background

The prevalence of overweight and obesity has increased rapidly worldwide [1]. Even traditionally ‘lean’ societies are beginning to experience the burdens associated with the increasing prevalence of obesity. The prevalence of overweight and obesity in Koreans continues to increase, with nearly 31.5% of adults over the age of 20 years currently considered overweight or obese [2]. The growing prevalence of overweight and obesity substantially contributes to the ongoing epidemic of type 2 diabetes and cardiovascular disease [3, 4]. Various weight-loss interventions, including diet and lifestyle modifications [5, 6], have shown efficacy in reducing body weight and improving metabolic risk factors, although the underlying mechanisms are not well understood.

Metabolomics is a technique that aims to identify and quantify the metabolome [7]. Metabolomics examines the human metabolome to detect metabolites (small molecules with a molecular weight less than 1500 Da) [8], metabolic pathways, and impairments in these pathways. Biofluids, tissues, or cellular extracts are analyzed. The use of metabolomics in nutrition research is increasing, and applications range from assessing novel biomarkers of dietary intake to intervention studies [9]. Using this technique to analyze the response to a dietary intervention generates valuable information concerning the effects of the prescribed diet on metabolic regulation [10–12].

The aim of this study was to examine changes in plasma metabolic profiles after a clinical intervention involving a 300 kcal/d intake reduction over a 12-week period in overweight adults. Assessments were made using ultra-performance liquid chromatography and mass spectrometry (UPLC-LTQ/Orbitrap MS), and the abdominal fat distribution was measured.

## Methods

### Study subjects

Study subjects were recruited through an advertisement in Seoul, Korea, from June 2015 to October 2016. Based on data screened by the Clinical Nutrigenetics/Nutrigenomics Laboratory at Yonsei University, subjects who were nondiabetic (fasting glucose < 126 mg/dL) and overweight [25 kg/m^2^ ≤ body mass index (BMI) < 30 kg/m^2^] were referred to the Department of Endocrinology at Yonsei University Severance Hospital. The subjects were rechecked their health and measured serum markers. Subjects who satisfied the study criteria were recommended for participation in the study. The inclusion criteria included being overweight and the absence of diabetes. The exclusion criteria were as follows: unstable body weight (body-weight change > 1 kg within 3 months before screening); hypertension; type 2 diabetes; cardiovascular, cerebrovascular, or thyroid disease; pregnancy or breast feeding; and consumption of medication that affected the body weight or energy expenditure. Additional exclusion criteria included acute or chronic infections; liver, kidney, or gastrointestinal disease; or any other acute/chronic disease requiring treatment. Finally, the aim of the study was carefully explained, and written informed consent was obtained from each participant prior to enrollment in the study. The Institutional Review Boards of Yonsei University and the Yonsei University Severance Hospital approved the study protocol, which complied with the Declaration of Helsinki.

### Study protocol and energy intake management

The participating subjects were allocated into two groups according to independently performed computer randomization (NCT03135132; http://clinicaltrials.gov/ct2/show/record/NCT03135132). The duration of the study was 12 weeks. The program goal for the LCD group was to lose at least 3% of their initial body weight. Over the study period, participants in the LCD group were subjected to calorie-restricted diets (approximately 300 kcal/d negative energy balance). Participants were recommended to remove 1/3 of a bowl of rice per meal per day to more easily achieve the 100 kcal deficits, because a bowl of rice has 300 kcal according to the food composition tables from the Rural Development Administration (8th Ed., 2011) of Korea. The normal dietary intake was recommended for the control group. The participants maintained a food diary that covered 3 days (2 weekdays and 1 weekend day), and good compliance with dietary interventions was defined as a reduction in the mean food intake value for 3 days of at least 300 kcal compared to baseline at the 12-week time point. The nutrient intake was determined and calculated as a mean value from the 3-day dietary record using the Computer-Aided Nutritional Analysis Program (CAN-pro 3.0, Korean Nutrition Society, Seoul, Korea). Additionally, physical activity was assessed using activity patterns [13], and the total energy expenditure (TEE) was calculated using the Harris-Benedict equation [14].

### Anthropometric parameters and body composition assessments

Body weight, height, BMI, waist circumference, and blood pressure (BP) were measured as previously described [15]. Each participant was interviewed using a structured questionnaire to assess their smoking and alcohol intake histories. The abdominal fat distribution was measured at the level of the L1 and L4 lumbar vertebrae via computed tomography (CT) using the GE Medical System HiSpeed Advantage® system (Milwaukee, WI, USA). Scanning was performed at a slice thickness of 1 mm at 200 mA and 120 kVp with a 48-cm field of view. The body composition, including the fat mass, lean body mass and fat percentage, was measured via dual-energy X-ray absorptiometry (DEXA; Discovery A; Hologic, Bedford, MA, USA). The data were analyzed using volume integration software (APEX 4.0.2 [13.4.1]; Hologic, Bedford, MA, USA).

### Blood collection

Blood samples were collected for the analysis of clinical characteristics after at least 12 h of overnight fasting. Venous blood specimens were collected in plain tubes and EDTA-treated whole blood tubes to separate the serum and plasma, respectively. The samples were centrifuged and stored at − 80 °C prior to analysis.

### Serum lipid profile, free fatty acid and glucose levels and homeostatic model assessment (HOMA) of insulin resistance (IR)

The triglyceride and total cholesterol levels were analyzed by enzymatic assays using a Hitachi 7600 autoanalyzer (Hitachi, Tokyo, Japan). The serum high-density lipoprotein (HDL) cholesterol concentrations were measured by selective inhibition with a Hitachi 7600 autoanalyzer. The low-density lipoprotein (LDL) cholesterol concentrations were calculated indirectly from the Friedwald formula, wherein LDL cholesterol = total cholesterol−[HDL cholesterol+(triglyceride/5)], for subjects with fasting triglyceride levels < 400 mg/dL. The levels of free fatty acids were obtained by an enzymatic assay using the acyl-CoA synthetase-acyl-CoA oxidase (ACS-ACOD) method with a Hitachi 7600 autoanalyzer. The serum glucose concentrations were determined using the hexokinase method on a Hitachi 7600 autoanalyzer. IR was calculated using HOMA using the following equation: HOMA-IR = [fasting insulin (μIU/mL) × fasting glucose (mg/dL)]/405.

### Plasma oxidized (ox)-LDL levels and lipoprotein-associated phospholipase A_2_ (Lp-PLA_2_) activity

The plasma ox-LDL level was assessed using an enzyme immunoassay (Mercodia AB, Uppsala, Sweden). The resulting change was monitored at 450 nm using a Wallac 1420 Victor^2^ Multi-Label Counter (PerkinElmer Life Sciences, Boston, MA, USA). Lp-PLA_2_ is also called platelet-activating factor acetylhydrolase (PAF-AH). The Lp-PLA_2_ activity was measured using a PAF-AH Activity Assay Kit (Biovision, Milpitas, CA, USA), which produces results based on a colorimetric shift, following the manufacturer’s instructions. The color reaction results were immediately measured at 412 nm using a VERSAmax Microplate Reader (Molecular Devices, Sunnyvale, CA, USA) in kinetic mode.

### Non-targeted metabolic profiling of plasma

#### UPLC-LTQ-Orbitrap XL MS analysis

Plasma extracts were prepared as previously described [15]. Chromatography was performed on a Thermo UPLC System (Ultimate 3000 BioRS; Dionex-Thermo Fisher Scientific, Bremen, Germany) coupled to a LTQ-Orbitrap-XL Mass Spectrometer (Thermo Fisher Scientific, Waltham, MA, USA). A total of 5 μL from each sample was separated using an Acquity UPLC-BEH-C18 column (2.1 × 50 mm, 1.7 μm; Waters, Milford, MA, USA), and the column oven was maintained at 50 °C. Mobile phase A was LC-MS-grade water (Fisher Scientific, Fair Lawn, NJ, USA) containing 0.1% formic acid, and mobile phase B was LC-MS-grade methanol (Fisher Scientific, Fair Lawn, NJ, USA) with 0.1% formic acid. The total flow rate was 0.4 mL/min, and the elution gradient (A:B, *v*/v) was changed from 100:0 to 0:100 in 15 min, maintained for 4 min and decreased to 100:0 for 2 min. The mass spectrometer was operated in ESI-positive mode with a mass resolution of 60,000. Full scan mass spectra were collected from 50 to 1000 *m/z*. The mass spectrometric parameters were set as follows; scan rate, 1 Hz; spray voltage, 5 kV; capillary voltage, 35 V; tube-lens voltage, 120 V; capillary temperature, 360 °C; flow rate of nitrogen sheath gas, 50 (arbitrary units); and flow rate of auxiliary gas, 5 (arbitrary units). A pooled quality control sample was made by mixing aliquots of each sample (Additional file 1: Figure S1). This sample was injected into every 5th sample to check the data quality and reproducibility (Additional file 1: Figure S2). In our QC data, the median relative standard deviation [RSD; also known as coefficient of variation (CV)] was 4.1%.

#### Data processing and putative identification of metabolites

The raw spectral data were aligned (Additional file 1: Table S1) and processed using the data analysis software SIEVE 2.2 (Thermo Fisher Scientific, Waltham, MA, USA). In this study, the retention time width parameter was set as 2.5 min, the *m/z* width was set as 5 ppm and the *m/z* range was set as 50–1000. The preliminary metabolites were identified from the following databases: Human Metabolome (www.hmdb.ca); Lipid MAPS (www.lipidmaps.org); Kyoto Encyclopedia of Genes and Genomes (KEGG) (www.genome.jp/kegg); MassBank (www.massbank.jp); and ChemSpider (www.chemspider.com). MS/MS was conducted to identify putative metabolites, and the applied collision energy was 20–55%. The resulting spectrometric data were compared with references from various MS/MS spectra databases.

### Statistical analyses

The SPSS version 23.0 software (IBM/SPSS, Chicago, IL, USA) was used for the statistical analysis. A logarithmic transformation was performed for the skewed variables. For descriptive purposes, the mean values were expressed as untransformed values and the mean ± standard error (SE). A two-tailed test with *p* < 0.05 was considered significant. For nominal variables, a Chi-squared test was performed. For continuous variables, an independent *t*-test was conducted to compare values between the control and LCD groups, and a paired *t*-test was used to compare values within each group. To correct the multiple comparison problem of the metabolites, a false discovery rate (FDR)-corrected *q* value was calculated with the R package “fdrtool” (http://cran.r-project.org/web/packages/fdrtool/). Pearson’s correlation coefficient was used to analyze relationships between variables. A heat map was created to visualize relationships among the clinical parameters and major metabolites.

A multivariate analysis was performed using SIMCA-P+ 14.0 (Umetrics, Inc., Umeå, Sweden). All data were Pareto scaled prior to the multivariate statistical analysis. The orthogonal projection to latent structures-discriminant analysis (OPLS-DA), which is a supervised classification tool, was used to analyze our models. The robustness and validity of the results were assessed using the *R*^*2*^*Y* and *Q*^*2*^*Y* parameters and cross validation-analysis of variance (CV-ANOVA).

## Results

### Effects of 12-week consumption of a LCD on anthropometric parameters, abdominal fat areas and biochemical parameters

After 12 weeks, 3 of the 100 subjects (3 LCD subjects) dropped out (two participants dropped out voluntarily, and one participant denied the CT measurement). Table 1 shows the body weights, BMIs, abdominal fat areas at L1 and L4, and biochemical parameters at baseline and 12 weeks in the control and LCD groups. At baseline, no significant differences between the two groups were observed in age, gender distribution, smoking, alcohol intake, body weight, BMI, whole fat area, visceral fat area (VFA), and subcutaneous fat area (SFA) at the L1 and L4 levels. After 12 weeks of treatment, the individuals in the LCD group showed significant reductions in their body weight (4.8% of their initial body weight), BMI, waist circumference, and total, visceral, and subcutaneous fat areas at the L1 and L4 levels compared to the baseline. When we compared the changes from the baseline between the control and LCD groups, significantly greater reductions were observed in body weight, BMI, waist circumference, and abdominal fat areas (VFA at L1 and L4 and SFA at L4) in the LCD group than in the control group. At 12 weeks, the LCD group had lower BMIs, waist circumferences, whole fat areas at L1 and L4, and VFA at L1 and L4 than the control group (Table 1).

**Table 1:** Effects of 12-week consumption of a low calorie diet (LCD) on body weight and abdominal fat areas by CT and biochemical parameters

	Control group (*n* = 50)		LCD group (*n* = 47)		*P* ^*a*^	*P* ^*b*^	*P* ^*c*^
	Baseline	Follow-up	Baseline	Follow-up			
Age (year)	41.0 ± 1.34		40.2	±1.57	0.673		
Male/Female *n*, (%)	14 (28.0)/36 (72.0)		13 (27.7)/34 (72.3)		0.970		
Cigarette smoker *n*, (%)	3 (6.0)		4 (8.5)		0.633		
Alcohol drinker *n*, (%)	30 (60.0)		24 (51.1)		0.376		
Weight (kg)	71.9 ± 1.24	72.1 ± 1.27	72.2 ± 1.36	68.8 ± 1.38^***^	0.887	0.074	
Change	0.21 ± 0.12		−3.44 ± 0.28				< 0.001
BMI (kg/m^2^)	27.1 ± 0.20	27.1 ± 0.21	27.2 ± 0.22	25.9 ± 0.25^***^	0.570	< 0.001	
Change	0.08 ± 0.04		−1.31 ± 0.11				< 0.001
Waist (cm)	91.8 ± 0.79	91.7 ± 0.72	90.6 ± 0.76	88.7 ± 0.76^***^	0.263	0.005	
Change	−0.16 ± 0.29		−1.90 ± 0.40				0.001
CT evaluation (L1)							
Whole fat area (cm^2^)	256.7 ± 7.87	260.6 ± 7.73	250.3 ± 8.00	233.4 ± 8.17^***^	0.575	0.017	
Change	3.97 ± 2.79		−16.9 ± 3.41				< 0.001
Visceral fat area (cm^2^)	117.9 ± 5.92	121.2 ± 5.80	109.2 ± 6.04	100.1 ± 5.63^***^	0.309	0.011	
Change	3.27 ± 2.29		−9.10 ± 2.40				< 0.001
Subcutaneous fat area (cm^2^)	138.8 ± 5.39	139.5 ± 5.22	141.1 ± 5.83	133.3 ± 5.50^***^	0.769	0.418	
Change	0.70 ± 1.05		−7.81 ± 1.94				< 0.001
CT evaluation (L4)							
Whole fat area (cm^2^)	307.9 ± 6.42	305.9 ± 6.35	300.7 ± 6.83	279.4 ± 6.77^***^	0.441	0.005	
Change	−2.06 ± 2.64		−21.3 ± 4.73				0.001
Visceral fat area (cm^2^)	93.4 ± 4.06	93.4 ± 3.92	90.9 ± 4.41	81.2 ± 4.39^**^	0.667	0.040	
Change	0.00 ± 1.51		−9.66 ± 2.79				0.003
Subcutaneous fat area (cm^2^)	214.5 ± 6.37	212.4 ± 6.12	209.8 ± 7.10	198.2 ± 5.81^*^	0.625	0.097	
Change	−2.05 ± 2.24		−11.6 ± 4.33				0.054
Systolic BP (mmHg)	119.0 ± 1.96	117.0 ± 1.66	121.1 ± 1.91	117.0 ± 1.87^*^	0.453	0.987	
Diastolic BP (mmHg)	73.7 ± 1.28	72.8 ± 1.09	74.7 ± 1.50	72.1 ± 1.29^*^	0.607	0.694	
Glucose (mg/dL)^*∮*^	88.5 ± 1.41	89.5 ± 1.26	86.4 ± 1.38	88.0 ± 1.45	0.288	0.378	
Insulin (μIU/dL)^*∮*^	15.6 ± 1.68	14.8 ± 1.61	13.5 ± 0.92	11.8 ± 0.82^*^	0.519	0.032	
HOMA-IR^*∮*^	3.42 ± 0.37	3.32 ± 0.43	2.91 ± 0.22	2.60 ± 0.21	0.392	0.028	
Free fatty acid (uEq/L)^*∮*^	617.2 ± 37.8	600.2 ± 34.6	614.8 ± 42.0	745.4 ± 50.2^*^	0.966	0.016	
Change	−17.0 ± 42.3		130.7 ± 58.1				0.041
Triglyceride (mg/dL)^*∮*^	125.1 ± 9.84	129.6 ± 9.18	111.9 ± 8.79	125.8 ± 30.4	0.230	0.445	
Total cholesterol (mg/dL)^*∮*^	206.9 ± 5.81	211.1 ± 5.80	195.0 ± 4.97	198.7 ± 5.36	0.151	0.132	
HDL cholesterol (mg/dL)^*∮*^	53.6 ± 1.53	54.9 ± 1.62	52.3 ± 1.83	53.4 ± 1.57	0.452	0.524	
LDL cholesterol (mg/dL)^*∮*^	128.7 ± 5.21	129.4 ± 5.08	120.2 ± 4.70	123.4 ± 4.59	0.264	0.430	
Oxidized LDL (U/L)^*∮*^	57.4 ± 2.53	54.9 ± 2.15	55.3 ± 2.61	51.2 ± 2.14	0.569	0.207	
Lp-PLA_2_ activity (nmol/ml/min)^*∮*^	28.1 ± 1.18	27.7 ± 1.29	27.2 ± 1.47	26.8 ± 1.50	0.603	0.364	

Mean ± SE.^∮^tested following logarithmic transformation. *P*^*a*^-values derived from a Chi-squared test or an independent *t*-test for the nominal or continuous variables, respectively, at baseline between groups. *P*^*b*^-values derived from an independent *t*-test at follow-up between groups. *P*^*c*^-values derived from an independent *t*-test at changed value between groups. ^*^p < 0.05, ^**^p < 0.01, and ^***^p < 0.001 derived from a paired *t*-test to compare baseline and follow-up within each group

At baseline, no significant differences between the two groups were observed in the systolic and diastolic blood pressure, glucose, insulin, HOMA-IR index, free fatty acids, serum lipid profiles, oxidized LDL, and Lp-PLA_2_ activity (Table 1). After 12 weeks of treatment, individuals in the LCD group exhibited significant reductions in systolic and diastolic blood pressure and serum insulin and a significant elevation in serum free fatty acids from baseline. Compared to the control group, the LCD group showed a significantly greater increase in the mean change in serum free fatty acids (*p* = 0.041). At 12 weeks, the LCD group showed lower insulin levels and HOMA-IR indices and higher free fatty acid levels than the control group (Table 1).

### Energy intake assessment

The estimated total caloric intakes at baseline were 2157.6 ± 35.9 kcal/d and 2174.3 ± 40.1 kcal/d in the control and LCD groups (*p* = 0.778), respectively. The LCD group had a greater reduction in the estimated total caloric intake than the control group (− 297.8 ± 6.12 kcal/d compared with 0.39 ± 6.99 kcal/d; *p* < 0.001). Moreover, significant decreases in the percentage of the total caloric intake from carbohydrates (59.7 ± 0.12% compared with 61.6 ± 0.12%, *p* < 0.001) and significant increases in the percentage of the total caloric intake from protein and fat (16.9 ± 0.05% compared with 15.9 ± 0.06%, *p* < 0.001; 23.7 ± 0.15% compared with 22.7 ± 0.17%, *p* < 0.001) were observed in the LCD group after 12 weeks of follow-up. However, the LCD group showed a decrease of actual amounts of major nutrient intake (g of carbohydrate, protein, and fat) at the follow-up compared to the baseline (all *p* < 0.001); and also showed reduced amounts of the major nutrient intake compared with the control group at the follow-up [carbohydrate, *p* < 0.001; protein, *p* = 0.006; and fat, *p* = 0.001]. Conversely, no significant differences were found in the control group (Additional file 1: Table S2). No significant differences were observed in the TEE and the percentage of participants who smoked and/or drank alcohol between baseline and the 12-week follow-up (data not shown).

### Plasma metabolic profiling using UPLC-LTQ Orbitrap mass spectrometry

#### Non-targeted metabolic pattern analysis

**Fig. 1:**
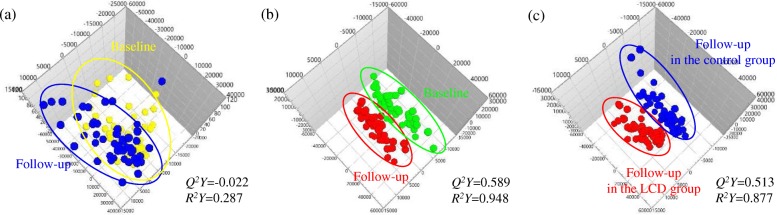
Identification of plasma metabolites with significantly altered levels. **a** Score plots from the OPLS-DA models in the control group (*n* = 50); comparison between baseline (yellow) and follow-up (blue). **b** Score plots from the OPLS-DA models in the low calorie diet (LCD) group (*n* = 47); comparison between baseline (green) and follow-up (red). **c** Score plots from the OPLS-DA models at follow-up; comparison between follow-up in the control (*n* = 50, blue) and LCD (*n* = 47, red) groups

**Fig. 2:**
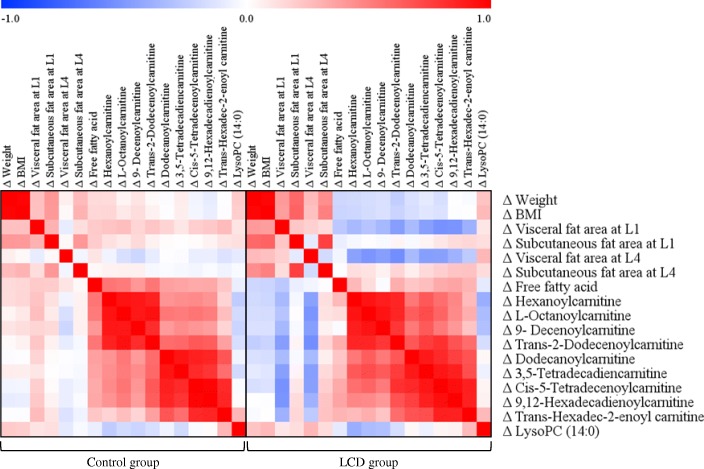
Correlation matrix among the changes (Δ) from baseline in clinical parameters and major metabolites in the control and low calorie diet (LCD) groups. Correlations were obtained by deriving a Pearson correlation coefficient. *Red* indicates a positive correlation, and *blue* indicates a negative correlation

The MS plasma metabolite data were obtained at baseline and the 12-week follow-up and analyzed using an OPLS-DA score plot. OPLS-DA is the most appropriate technique to search for metabolic profiles that define the characteristics of LCD-induced mild weight loss because the predictive component in the OPLS-DA describes the treatment effects after excluding the variance between samples in the same group [16]. OPLS-DAs were conducted for the following three combinations of groups: (1) comparison between the baseline and 12-week follow-up levels of the control group (*n* = 50) (Fig. 1a); (2) comparison between the baseline and 12-week follow-up levels of the LCD group (*n* = 47) (Fig. 1b); and (3) comparison between the 12-week follow-up levels of the control (*n* = 50) and LCD groups (*n* = 47) (Fig. 1c).

The quality of each OPLS-DA model was examined using the *R*^2^ and *Q*^2^ values to confirm that the models were not over-fitted and to evaluate the predictive ability of each model. *R*^2^ represents the goodness-of-fit parameter and is defined as the proportion of variance in the data described by the model. *Q*^2^ represents the predictive ability parameter and is defined as the proportion of variance in the data predicted by the model. *R*^2^ and *Q*^2^ values greater than 0.5 indicate a high-quality OPLS-DA model [17]. The two-component OPLS-DA scatter plots of the plasma metabolites for the first combination of groups did not show distinct clustering or a clear separation of participants in the control group at baseline or the 12-week follow-up assessment (*R*^2^*Y* = 0.287, *Q*^2^*Y* = − 0.0219). However, these analyses revealed that the second and third models (Fig. 1) displayed greater than 87% goodness-of-fit (*R*^2^*Y* = 0.948 and *R*^2^*Y* = 0.877 for the second and third combinations of groups, respectively) and a predictive ability greater than 51% (*Q*^2^*Y* = 0.589 and *Q*^2^*Y* = 0.513 for the second and third combinations of groups, respectively). Therefore, the second and third OPLS-DA models in this study were well-fitted and displayed an acceptable predictive ability. This result clearly shows that plasma metabolomics profiles can distinguish groups based on a LCD, changes in LCD-induced biochemical characteristics, or both parameters.

#### Putative identification of plasma metabolites

The variables that contributed to the separation between the groups were selected according to the variable importance in the projection (VIP) method; VIP values > 1.0 indicate a high relevance for differences between sample groups. A total of 225 variables had VIP values > 1.0, of which 43 were previously identified metabolites, and the rest were unknown variables. The results are shown in Table 2. No significant differences were observed in the metabolites at baseline between the control and LCD groups. At the 12-week follow-up assessment, all 43 metabolites from the control group were not significantly different from baseline. In the LCD group, a significant increase was observed in the hexanoylcarnitine (C6), L-octanoylcarnitine (C8), 9-decenoylcarnitine (C10:1), trans-2-dodecenoylcanitine (C12:1), dodecanoylcarnitine (C12), 3,5-tetradecadiencarnitine (C14:2), cis-5-tetradecenoylcarnitine (C14:1), 9,12-hexadecadienoylcarnitine (C16:2), and 9-hexadecenoylcarnitne (C16:1) levels, and a significant decrease was observed in the lysophosphatidylcholine (lysoPC) level (14:0) at the 12-week follow-up assessment (Table 2). Next, we compared the plasma metabolite changes from baseline between the control and LCD groups. The LCD group showed significantly greater increases in hexanoylcarnitine (*q* = 0.033), L-octanoylcarnitine (*q* = 0.033), trans-2-dodecenoylcanitine (*q* = 0.033), and 3,5-tetradecadiencarnitine (*q* = 0.037) than the control group. Additionally, the LCD group exhibited a trend toward increases in 9-decenoylcarnitine (*q* = 0.051) and cis-5-tetradecenoylcarnitine (*q* = 0.076). At the 12-week follow-up assessment, the LCD group had significantly higher peak intensities of hexanoylcarnitine (*q* = 0.001), L-octanoylcarnitine (*q* = 0.001), 9-decenoylcarnitine (*q* = 0.002), trans-2-dodecenoylcanitine (*q* = 0.001), dodecanoylcarnitine (*q* = 0.003), 3,5-tetradecadiencarnitine (*q* = 0.002), cis-5-tetradecenoylcarnitine (*q* = 0.003), 9,12-hexadecadienoylcarnitine (*q* = 0.006), 9-hexadecenoylcarnitne (*q* = 0.033), 11Z-octadecenylcarnitine (*q* = 0.035) and lysoPC (20:4) (*q* = 0.002) than the control group (Table 2).

**Table 2:** Putative identification of plasma metabolites in the control and low calorie diet (LCD) groups at the baseline and follow-up assessments

Identified metabolite	Molecular formula	*m/z* [M + H]	Normalized peak intensities				VIP		*q* ^*a*^	*q* ^*b*^	*q* ^*c*^
			Control group (*n* = 50)		LCD group (*n* = 47)		Baseline vs. follow-up in the weight loss group	Placebo follow-up vs. test follow-up			
			Baseline	Follow-up	Baseline	Follow-up					
Leucine	C_6_H_13_NO_2_	132.101	69,199,518 ± 1,836,711	67,420,761 ± 1,582,674	68,873,642 ± 2,357,457	66,991,101 ± 1,680,467	2.464	1.909	0.932	0.596	
Cinnamic acid	C_9_H_8_O_2_	149.060	46,918 ± 1558	498,417 ± 451,353	53,897 ± 5095	49,790 ± 3202	0.044	1.194	0.863	0.418	
Phenylalanine	C_9_H_11_NO_2_	166.086	36,208,763 ± 997,849	36,755,754 ± 813,575	35,402,342 ± 874,666	37,279,370 ± 1,022,374	3.021	1.614	0.891	0.543	
Tyrosine	C_9_H_11_NO_3_	182.081	22,155,726 ± 651,812	22,600,874 ± 639,963	21,257,580 ± 596,338	22,640,873 ± 739,425	2.616	1.324	0.863	0.626	
3-Indolepropionic acid	C_11_H_11_NO_2_	190.086	4,002,006 ± 1,272,195	4,041,927 ± 1,236,682	1,271,269 ± 134,543	1,227,563 ± 120,655	0.515	2.978	0.863	0.229	
Dodecanamide	C_12_H_25_NO	200.201	1,558,068 ± 258,678	1,863,395 ± 378,657	1,581,902 ± 282,781	1,393,313 ± 210,938	1.092	1.559	0.934	0.411	
Tryptophan	C_11_H_12_N_2_O_2_	205.097	39,197,768 ± 1,458,101	39,839,464 ± 1,415,489	38,501,638 ± 1,871,339	39,134,171 ± 1,617,069	2.595	2.168	0.920	0.562	
3-Hydroxydodecanoic acid	C_12_H_24_O_3_	217.179	3,246,500 ± 518,641	3,723,180 ± 750,057	3,585,802 ± 631,353	4,089,347 ± 605,082	1.753	1.774	0.910	0.550	
Palmitic amide	C_16_H_33_NO	256.263	13,751,407 ± 1,082,041	15,138,462 ± 1,359,736	15,340,611 ± 1,075,051	15,153,308 ± 1,147,945	2.330	2.499	0.863	0.632	
Hexanoylcarnitine	C_13_H_25_NO_4_	260.186	608,614 ± 30,592	612,447 ± 29,061	634,093 ± 38,818	909,940 ± 57,087^***^	1.368	1.537	0.900	0.001	
Change			3833 ± 41,113		275,848 ± 59,070						0.033
Oleamide	C_18_H_35_NO	282.278	78,372,127 ± 4,740,634	82,082,837 ± 6,769,412	89,539,229 ± 5,022,276	79,086,450 ± 4,856,087	7.485	5.628	0.863	0.554	
2-Octenoylcarnitine	C_15_H_27_NO_4_	286.201	3,403,461 ± 209,099	3,295,980 ± 194,746	3,519,700 ± 248,614	4,083,782 ± 298,458	1.484	1.750	0.915	0.236	
L-Octanoylcarnitine	C_15_H_29_NO_4_	288.216	2,142,223 ± 118,455	2,099,293 ± 101,216	2,312,360 ± 147,156	3,279,385 ± 233,253^***^	2.502	3.068	0.867	0.001	
Change			−42,930 ± 139,259		967,025 ± 228,837						0.033
9-Decenoylcarnitine	C_17_H_31_NO_4_	314.232	3,402,770 ± 186,196	3,415,091 ± 162,320	3,556,295 ± 232,420	4,836,086 ± 291,858^***^	2.900	3.292	0.900	0.002	
Change			12,322 ± 222,399		1,279,791 ± 300,197						0.051
Trans-2-Dodecenoylcarnitine	C_19_H_35_NO_4_	342.263	1,598,708 ± 125,856	1,497,283 ± 83,951	1,650,280 ± 124,122	2,402,866 ± 161,419^***^	2.341	3.039	0.920	0.001	
Change			−101,426 ± 138,742		752,586 ± 185,951						0.033
Dodecanoylcarnitine	C_19_H_37_NO_4_	344.279	1,424,275 ± 153,492	1,165,937 ± 87,541	1,420,237 ± 157,620	2,043,414 ± 189,930^*^	1.938	2.879	0.936	0.003	
Tetracosahexaenoic acid	C_24_H_36_O_2_	357.278	1,658,005 ± 271,155	1,718,916 ± 200,221	2,038,912 ± 358,879	1,351,020 ± 128,186	2.073	0.735	0.872	0.372	
3,5-Tetradecadiencarnitine	C_21_H_37_NO_4_	368.279	1,131,603 ± 122,534	968,845 ± 76,487	1,066,107 ± 90,189	1,643,966 ± 135,858^**^	2.061	2.606	0.909	0.002	
Change			−162,758 ± 134,815		577,859 ± 155,842						0.037
Cis-5-Tetradecenoylcarnitine	C_21_H_39_NO_4_	370.294	1,706,081 ± 185,912	1,583,864 ± 134,276	1,780,591 ± 160,767	2,608,263 ± 212,663^**^	2.324	3.169	0.919	0.003	
Change			−122,217 ± 178,687		827,672 ± 238,593						0.076
Tetradecanoylcarnitine	C_21_H_41_NO_4_	372.310	196,602 ± 18,151	201,544 ± 23,048	196,335 ± 21,232	261,601 ± 68,648	0.674	1.145	0.937	0.431	
9,12-Hexadecadienoylcarnitine	C_23_H_41_NO_4_	396.310	200,840 ± 23,812	179,743 ± 15,299	216,362 ± 22,755	305,619 ± 28,345^**^	0.695	1.057	0.905	0.006	
9-Hexadecenoylcarnitne	C_23_H_43_NO_4_	398.326	805,349 ± 78,646	764,402 ± 59,953	879,547 ± 75,817	1,082,378 ± 78,777^*^	1.025	1.516	0.886	0.033	
L-Palmitoylcarnitine	C_23_H_45_NO_4_	400.342	2,659,345 ± 235,470	2,492,746 ± 158,183	2,964,365 ± 212,908	3,089,189 ± 173,630	0.971	1.874	0.863	0.150	
Linoleyl carnitine	C_25_H_45_NO_4_	424.341	3,432,241 ± 258,751	3,448,289 ± 251,617	3,974,869 ± 282,822	3,997,750 ± 239,882	0.991	1.479	0.863	0.365	
11Z-Octadecenylcarnitine	C_25_H_47_NO_4_	426.357	4,028,850 ± 366,572	3,777,116 ± 227,107	4,624,006 ± 332,692	4,855,270 ± 247,842	1.175	2.586	0.863	0.035	
Stearoylcarnitine	C_25_H_49_NO_4_	428.373	788,773 ± 92,558	682,655 ± 60,559	1,031,733 ± 153,656	854,050 ± 60,947	1.043	0.898	0.863	0.287	
Glycoursodeoxycholic acid	C_26_H_43_NO_5_	450.321	50,921 ± 7574	57,755 ± 8421	98,290 ± 41,323	101,322 ± 26,695	1.006	0.535	0.863	0.369	
LysoPC (14:0)	C_22_H_46_NO_7_P	468.307	10,040,884 ± 764,081	10,401,952 ± 763,675	10,689,233 ± 718,261	8,424,696 ± 507,725^**^	3.674	3.330	0.890	0.248	
LysoPE (18:2)	C_23_H_44_NO_7_P	478.292	4,959,398 ± 371,744	5,069,861 ± 286,366	4,805,440 ± 259,491	4,682,920 ± 231,774	0.879	1.335	0.917	0.414	
LysoPC (P-16:0)	C_24_H_50_NO_6_P	480.344	4,110,993 ± 341,788	3,970,485 ± 280,525	4,664,875 ± 339,451	4,743,238 ± 340,113	1.256	1.874	0.863	0.336	
LysoPC (15:0)	C_23_H_48_NO_7_P	482.324	5,290,855 ± 402,662	5,345,146 ± 372,812	5,878,370 ± 381,664	5,434,814 ± 307,358	1.512	1.203	0.863	0.596	
LysoPC (16:1)	C_24_H_48_NO_7_P	494.322	33,323,736 ± 2,486,845	32,403,948 ± 2,140,796	34,631,912 ± 2,132,250	32,861,351 ± 1,872,070	2.379	2.239	0.912	0.601	
LysoPC (16:0)	C_24_H_50_NO_7_P	496.338	138,949,923 ± 4,569,604	135,185,761 ± 3,936,092	144,202,989 ± 4,066,681	141,523,884 ± 3,297,945	4.511	4.869	0.872	0.402	
LysoPE (20:5)	C_25_H_42_NO_7_P	500.276	1,347,355 ± 89,611	1,464,681 ± 78,217	1,303,582 ± 81,981	1,218,180 ± 62,061	0.525	1.050	0.915	0.174	
LysoPC (17:0)	C_25_H_52_NO_7_P	510.354	6,009,897 ± 513,695	6,076,807 ± 518,215	6,665,983 ± 467,898	6,616,976 ± 468,650	1.427	1.666	0.863	0.449	
LysoPC (18:4)	C_26_H_46_NO_7_P	516.307	2,447,450 ± 258,665	1,910,581 ± 195,850	2,458,064 ± 237,607	2,669,297 ± 263,124	1.055	2.093	0.936	0.204	
LysoPC (18:3)	C_26_H_48_NO_7_P	518.323	15,179,171 ± 684,691	13,872,739 ± 565,055	14,630,190 ± 688,150	14,496,638 ± 693,728	1.083	1.961	0.896	0.468	
LysoPC (18:2)	C_26_H_50_NO_7_P	520.338	126,463,727 ± 4,429,933	119,011,941 ± 4,540,519	131,062,544 ± 5,257,011	134,980,434 ± 5,451,322	2.968	4.143	0.886	0.222	
LysoPC (18:1)	C_26_H_52_NO_7_P	522.353	46,404,015 ± 1,970,134	46,855,407 ± 1,807,203	49,728,198 ± 1,732,770	50,063,092 ± 1,565,998	2.799	3.721	0.863	0.393	
LysoPC (20:5)	C_28_H_48_NO_7_P	542.322	21,296,265 ± 1,180,839	23,129,472 ± 1,427,868	22,695,421 ± 1,382,332	21,128,248 ± 1,302,925	1.824	1.702	0.879	0.414	
LysoPC (20:4)	C_28_H_50_NO_7_P	544.338	23,325,452 ± 773,358	22,972,855 ± 648,846	25,773,359 ± 759,935	26,979,655 ± 665,976	2.371	5.282	0.863	0.002	
LysoPC (20:3)	C_28_H_52_NO_7_P	546.353	11,404,669 ± 384,062	11,060,195 ± 353,164	11,453,253 ± 341,767	11,416,355 ± 314,693	1.135	1.377	0.933	0.455	
LysoPC (22:6)	C_30_H_50_NO_7_P	568.338	6,497,939 ± 384,463	6,751,525 ± 359,584	7,097,799 ± 397,002	7,370,800 ± 349,895	1.231	1.528	0.863	0.401	

Mean ± SE. VIP is the variable importance in the projection. The *q*^*a*^-value is the adjusted *p*-value derived from the independent *t*-test in the baseline and controls based on the false discovery rate (FDR). The *q*^*b*^-value is the adjusted *p*-value derived from the independent *t*-test in the follow-up and controls based on the FDR. The *q*^*c*^-value is the adjusted *p*-value derived from the independent *t*-test in the changed values and controls based on the FDR

### The relationship between changes in the body weight, abdominal fat distribution, free fatty acids and major metabolites

**Fig. 3:**
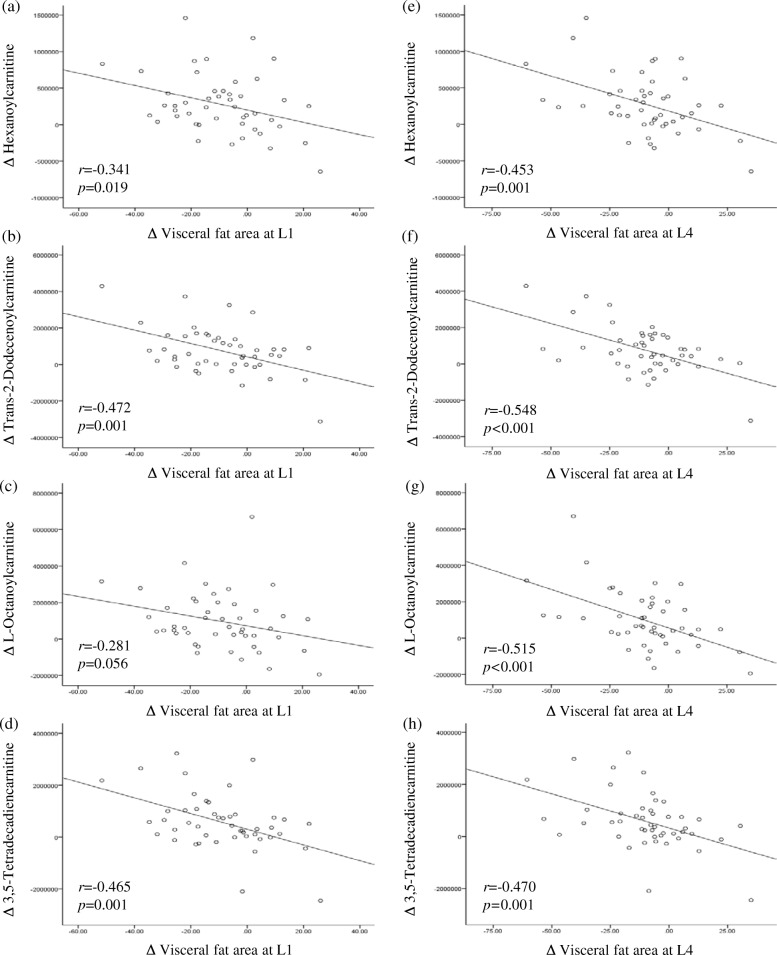
Correlation scatter plots of changes (Δ) from baseline in the major acylcarnitines and visceral fat areas at L1 and L4 in the low calorie diet (LCD) group. **a**, **b**, **c**, **d** Correlations between Δ hexanoylcarnitine, Δ L-octanoylcarnitine, Δ trans-2-dodecenoylcarnitine, Δ 3,5-tetradecadiencarnitine and Δ visceral fat area at L1, respectively. **e**, **f**, **g**, **h** Correlations between Δ hexanoylcarnitine, Δ L-octanoylcarnitine, Δ trans-2-dodecenoylcarnitine, Δ 3,5-tetradecadiencarnitine and Δ visceral fat area at L4, respectively

Figure 2 shows the correlations between the changes from baseline in body weight, BMI, VFA and SFA at L1, VFA and SFA at L4, serum free fatty acids, and the major metabolites that were significantly different between baseline and the 12-week follow-up in the LCD group. Figure 3 shows correlation scatter plots of changes from baseline in the major acylcarnitines (ACs) and the VFA at L1 and L4 in the LCD group. The mean changes in the major ACs were significantly different between the control and LCD groups. In the LCD group, changes in the VFA at L1 were strongly negatively correlated with changes in hexanoylcarnitine, trans-2-dodecenoylcanitine and 3,5-tetradecadiencarnitine. Similarly, changes in the VFA at L4 were strongly negatively correlated with changes in hexanoylcarnitine, trans-2-dodecenoylcanitine, L-octanoylcarnitine, and 3,5-tetradecadiencarnitine (Fig. 3).

## Discussion

This study was performed to identify important plasma metabolites that were affected by LCD-induced mild weight loss and changes in metabolic characteristics from baseline in overweight subjects. These data could yield insights into the mechanisms underlying mild weight loss. For the overweight participants of this study, a 12-week LCD intervention resulted in a weight reduction of 4.8%, an 8.3% reduction in the VFA at L1 and a 10.6% decrease in the VFA at L4. We showed that mild weight loss from a 12-week LCD resulted in significantly greater increases in the plasma hexanoylcarnitine (C6), L-octanoylcarnitine (C8), trans-2-dodecenoylcanitine (C12:1), and 3,5-tetradecadiencarnitine (C14:2) levels compared to the control group. Additionally, the changes in these ACs strongly negatively correlated with changes in the VFA at L1 and/or L4 in the LCD group. However, no significant association between changes in the VFA and these ACs was observed in the control group. Therefore, the LCD-induced visceral fat reduction is partially associated with increases in medium- or long-chain ACs.

ACs are intermediate oxidative metabolites that comprise a fatty acid esterified to a carnitine molecule, and they are excellent indicators of altered fatty acid oxidation [18]. ACs are generated by both mitochondrial and peroxisomal enzymes, including the carnitine palmitoyltransferase 1 (CPT1) and CPT2 enzymes, for the transportation of long-chain fatty acids (LCFAs) across the mitochondrial membrane for β-oxidation [19]. A recent study involving plasma metabolomic profiling of overweight individuals with a high VFA showed higher plasma levels of medium- and long-chain ACs compared to individuals with low VFA matched for age, gender, and BMI [20]. Additionally, this study suggested that the chronic lipid surplus from the VFA in overweight individuals with a high VFA was likely associated with substantial increases in plasma medium- and long-chain ACs, which are positively correlated with atherogenic traits [20]. However, in this study, the plasma medium- and long-chain ACs and serum free fatty acids increased after 12-week LCD-induced mild weight loss with a significant VFA reduction, whereas serum insulin decreased from baseline. These results indicate that an increase in medium- and long-chain ACs in the LCD group may result from the higher load of free fatty acids from the lipolysis of visceral fat. As a result, serum free fatty acids released from visceral fat could drive the fatty acid oxidation rates and generate ACs. This result is similar to a recent finding of an increase in several AC species in association with weight loss in obese human subjects despite improvements in insulin sensitivity [21]. These results support the suggestion that the plasma AC levels during weight loss may be driven by the lipolysis rate and the improved efflux from visceral adipocytes, potentially resulting from improved carnitine acetyltransferase activity and not by deranged mitochondrial fatty acid oxidation.

In contrast to the VFA, correlations between changes in SFA and ACs were not observed in the LCD group. This difference supports previous finding that visceral adipocytes are more metabolically active and more sensitive to lipolysis and thus have a greater capacity to generate free fatty acids than subcutaneous adipose tissue [22]. Indeed, in this study, the LCD group with visceral fat reduction showed a greater increase in serum free fatty acids than the control group without significant changes in the VFA. Additionally, the greater reductions in the VFA (measured by percentage) is in agreement with the finding that visceral adipose tissue is more sensitive to weight reduction than subcutaneous adipose tissue [22].

Meanwhile, diet composition can affect serum lipids and fatty acid chain in ACs. In this study, a composition of major nutrients in the LCD group was changed during the intervention (Additional file 1: Table S2). Thus, we analyzed correlations between changes in actual amount of each major nutrient intake (g of carbohydrate, protein, and fat) and changes in major ACs (hexanoylcarnitine, L-octanoylcarnitine, trans-2-dodecenyolcarnitine, and 3,5-tetradecadiencarnitine) in the control and LCD groups to verify that which change was associated with alteration of plasma AC profiles. In the control group, any significant correlations were not observed (data not shown). In the LCD group, significant negative correlations were observed between changes in the amount of carbohydrate intake (g) and changes in hexanoylcarnitine (*r* = − 0.337, *p* = 0.020), L-octanoylcarnitine (*r* = − 0.333, *p* = 0.022), and trans-2-dodecenyolcarnitine (*r* = − 0.350, *p* = 0.016), respectively. Surprisingly, the reduced amount of fat intake in the LCD group was not correlated with changes in AC profiles (data not shown). We thought that in the LCD group, because 1) the reduction of carbohydrate intake largely contributed to decrease in total calorie intake and 2) the calorie intake restriction caused weight loss (lipolysis), changes in the amount of carbohydrate intake showed correlations with the ACs. In other words, changes in nutrition composition might not greatly affect plasma AC profile changes in this study; whereas calorie restriction-induced weight loss with a significant VFA reduction might result in plasma AC profile changes as we discussed above.

An increase in the plasma lysoPC levels has been reported in obesity [23]. Hiemerl et al. reported that most lysoPC species in the plasma decreased after a 3-month rapid weight loss period (BMI change: − 6.62 kg/m^2^). However, in this study, no significant differences in mean changes in the lysoPC species were found between the control and LCD groups. Schwab et al. also found that the lysoPCs were not altered by weight reduction. LysoPC is a major component of oxidized LDL [24, 25]. Phospholipase A_2_ (PLA_2_), including secretory PLA_2_ and lipoprotein-associated PLA_2_ (Lp-PLA_2_), hydrolyzes PC, which simultaneously generates one molecule of lysoPC and one molecule of fatty acid [26, 27]. Therefore, lysoPC accumulation reflects increased production through PLA_2_-catalyzed PC hydrolysis. However, in this study, no significant differences were found in the mean changes in oxidized LDL and Lp-PLA_2_ activity between the control and LCD groups. Thus, the lack of changes in the lysoPC levels may partially be explained by the weight reduction observed in this study, which was not very dramatic, instead of the complexity of lysoPC metabolism.

This study has some limitations. First, we specifically focused on overweight individuals without diabetes. Therefore, our data cannot be generalized to obese individuals or diabetic patients. Second, dietary intake was based on self-reports obtained from weighed food. However, measurement errors from self-reported dietary intake and lifestyle variables have been shown to be relatively small [28, 29]. Third, although a large number of metabolites have been detected by UPLC-LTQ-Orbitrap MS in this study, most of the metabolites are currently unidentified. Large databases of endogenous biomolecules have not yet been constructed for use with LC-MS-based techniques for metabolomics research [30]. Despite these limitations, our approach using UPLC-LTQ-Orbitrap MS-based metabolomics and multivariate data analysis revealed significantly greater increases in the plasma hexanoylcarnitine, L-octanoylcarnitine, trans-2-dodecenoylcanitine, and 3,5-tetradecadiencarnitine levels in the LCD group during the 12-week mild caloric restriction period compared to the control group. The strong negative correlations between the changes in these ACs and changes in the VFA at L1 and/or L4 in the LCD group suggest that the LCD-induced visceral fat reduction is partially associated with an increase in medium- or long-chain ACs. Additionally, with the greater increase in serum free fatty acids observed in the LCD group compared to the control group, the level of plasma ACs in weight loss could be driven by the rate of lipolysis as well as improved efflux from visceral adipocytes.

## Conclusion

Our results suggested that mild weight loss from 12-week calorie restriction increased the plasma levels of medium- and long-chain ACs. These changes were coupled with a decrease in VFA and an increase in free fatty acids. Unlike a recent study [20], this study verifies other mechanism on increase of medium- and long-chain ACs in overweight subjects; thus, when we interpret metabolic data of overweight subjects, we should consider subjects’ recent VFA changes.

## Additional file

Additional file 1: Supplementary data. **Figure S1**. An example of chromatogram of a QC sample. **Figure S2**. PCA with the QC samples. **Table S1**. Alignment score of the sampels. **Table S2**. Comparison of major nutrients' composition between the control and LCD groups. (DOCX 104 kb)

## Abbreviations

ACs: Acylcarnitines
BMI: Body mass index
BP: Blood pressure
CPT: Carnitine palmitoyltransferase
CT: Computed tomography
DEXA: Dual-energy X-ray absorptiometry
FDR: False discovery rate
HDL: High-density lipoprotein
HOMA: Homeostatic model assessment
IR: Insulin resistance
LCD: Low calorie diet
LDL: Low-density lipoprotein
Lp-PLA_2_: Lipoprotein-associated phospholipase A_2_
OPLS-DA: Orthogonal projection to latent structures-discriminant analysis
Ox-: Oxidized
PAF-AH: Platelet-activating factor acetylhydrolase
SE: Standard error
SFA: Subcutaneous fat area
TEE: Total energy expenditure
UPLC: Ultra-performance liquid chromatography
VFA: Visceral fat area
VIP: Variable importance in the projection
