# Supplementary material for: Metabolomics identifies increases in the acylcarnitine profiles in the plasma of overweight subjects in response to mild weight loss: a randomized, controlled design study
Source: Lipids Health Dis. 2018 Oct 15;17:237. doi: 10.1186/s12944-018-0887-1 (PMC6190541; doi:10.1186/s12944-018-0887-1)
Supplement: Supplementary file 1 — Supplementary data. Figure S1. An example of chromatogram of a QC sample. Figure S2. PCA with the QC samples. Table S1. Alignment score of the sampels. Table S2. Comparison of major nutrients' composition between the control and LCD groups. (DOCX 104 kb) [file 12944_2018_887_MOESM1_ESM.docx]

**Additional file 1**

**Metabolomics identifies increases in the acylcarnitine profiles in the plasma of overweight subjects in response to mild weight loss: a randomized, controlled design study**


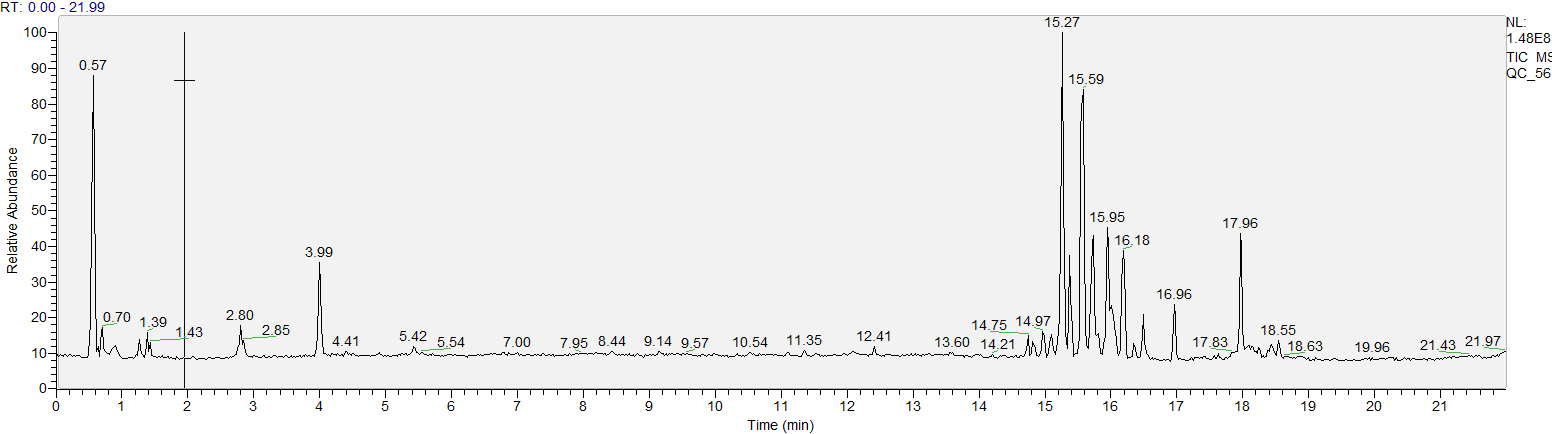


**Figure S1. An example of chromatogram of a QC sample**


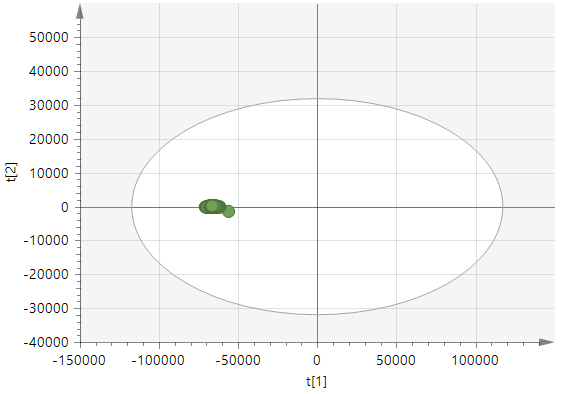


**Figure S2. PCA with the QC samples**

**Table S1. Alignment score of the samples**

| **ID** | **Align score** | **ID** | **Align score** | **ID** | **Align score** |
| --- | --- | --- | --- | --- | --- |
| Sample001 | 0.908 | Sample047 | 0.946 | Sample093 | 0.969 |
| Sample002 | 0.917 | Sample048 | 0.957 | Sample094 | 0.972 |
| Sample003 | 0.913 | Sample049 | 0.953 | Sample095 | 0.958 |
| Sample004 | 0.921 | Sample050 | 0.964 | Sample096 | 0.966 |
| Sample005 | 0.964 | Sample051 | 0.915 | Sample097 | 0.969 |
| Sample006 | 0.964 | Sample052 | 0.929 | Sample098 | 0.942 |
| Sample007 | 0.952 | Sample053 | 0.915 | Sample099 | 0.943 |
| Sample008 | 0.969 | Sample054 | 0.900 | Sample100 | 0.959 |
| Sample009 | 0.955 | Sample055 | 0.870 | Sample101 | 0.916 |
| Sample010 | 0.969 | Sample056 | 0.902 | Sample102 | 0.903 |
| Sample011 | 0.962 | Sample057 | 0.964 | Sample103 | 0.908 |
| Sample012 | 0.951 | Sample058 | 0.970 | Sample104 | 0.904 |
| Sample013 | 0.949 | Sample059 | 0.961 | Sample105 | 0.892 |
| Sample014 | 0.953 | Sample060 | 0.966 | Sample106 | 0.910 |
| Sample015 | 0.963 | Sample061 | 0.960 | Sample107 | 0.956 |
| Sample016 | 0.966 | Sample062 | 0.960 | Sample108 | 0.915 |
| Sample017 | 0.950 | Sample063 | 0.961 | Sample109 | 0.949 |
| Sample018 | 0.969 | Sample064 | 0.967 | Sample110 | 0.941 |
| Sample019 | 0.960 | Sample065 | 0.968 | Sample111 | 0.938 |
| Sample020 | 0.929 | Sample066 | 0.962 | Sample112 | 0.929 |
| Sample021 | 0.939 | Sample067 | 0.958 | Sample113 | 0.923 |
| Sample022 | 0.932 | Sample068 | 0.954 | Sample114 | 0.913 |
| Sample023 | 0.961 | Sample069 | 0.965 | Sample115 | 0.931 |
| Sample024 | 0.934 | Sample070 | 0.965 | Sample116 | 0.932 |
| Sample025 | 0.934 | Sample071 | 0.969 | Sample117 | 0.933 |
| Sample026 | 0.931 | Sample072 | 0.960 | Sample118 | 0.922 |
| Sample027 | 0.926 | Sample073 | 0.957 | Sample119 | 0.925 |
| Sample028 | 0.918 | Sample074 | 0.955 | Sample120 | 0.903 |
| Sample029 | 0.947 | Sample075 | 0.952 | Sample121 | 0.939 |
| Sample030 | 0.964 | Sample076 | 0.930 | Sample122 | 0.926 |
| Sample031 | 0.968 | Sample077 | 0.940 | Sample123 | 0.952 |
| Sample032 | 0.935 | Sample078 | 0.962 | Sample124 | 0.938 |
| Sample033 | 0.957 | Sample079 | 0.933 | Sample125 | 0.951 |
| Sample034 | 0.965 | Sample080 | 0.950 | Sample126 | 0.932 |
| Sample035 | 0.962 | Sample081 | 0.941 | Sample127 | 0.914 |
| Sample036 | 0.961 | Sample082 | 0.926 | Sample128 | 0.892 |
| Sample037 | 0.959 | Sample083 | 0.936 | Sample129 | 0.918 |
| Sample038 | 0.969 | Sample084 | 0.939 | Sample130 | 0.918 |
| Sample039 | 0.969 | Sample085 | 0.954 | Sample131 | 0.907 |
| Sample040 | 0.962 | Sample086 | 0.958 | Sample132 | 0.881 |
| Sample041 | 0.930 | Sample087 | 0.953 | Sample133 | 0.903 |
| Sample042 | 0.939 | Sample088 | 0.963 | Sample134 | 0.899 |
| Sample043 | 0.943 | Sample089 | 0.964 | Sample135 | 0.884 |
| Sample044 | 0.941 | Sample090 | 0.965 | Sample136 | 0.909 |
| Sample045 | 0.939 | Sample091 | 0.964 | Sample137 | 0.900 |
| Sample046 | 0.955 | Sample092 | 0.967 | Sample138 | 0.956 |
| **ID** | **Align score** | **ID** | **Align score** |  |  |
| Sample139 | 0.895 | Sample186 | 0.913 |  |  |
| Sample140 | 0.906 | Sample187 | 0.908 |  |  |
| Sample141 | 0.947 | Sample188 | 0.912 |  |  |
| Sample142 | 0.941 | Sample189 | 0.953 |  |  |
| Sample143 | 0.935 | Sample190 | 0.937 |  |  |
| Sample144 | 0.942 | Sample191 | 0.938 |  |  |
| Sample145 | 0.933 | Sample192 | 0.944 |  |  |
| Sample146 | 0.945 | Sample193 | 0.950 |  |  |
| Sample147 | 0.938 | Sample194 | 0.943 |  |  |
| Sample148 | 0.923 |  |  |  |  |
| Sample149 | 0.922 |  |  |  |  |
| Sample150 | 0.913 |  |  |  |  |
| Sample151 | 0.925 |  |  |  |  |
| Sample152 | 0.924 |  |  |  |  |
| Sample153 | 0.936 |  |  |  |  |
| Sample154 | 0.936 |  |  |  |  |
| Sample155 | 0.936 |  |  |  |  |
| Sample156 | 0.952 |  |  |  |  |
| Sample157 | 0.948 |  |  |  |  |
| Sample158 | 0.935 |  |  |  |  |
| Sample159 | 0.916 |  |  |  |  |
| Sample160 | 0.925 |  |  |  |  |
| Sample161 | 0.932 |  |  |  |  |
| Sample162 | 0.927 |  |  |  |  |
| Sample163 | 0.931 |  |  |  |  |
| Sample164 | 0.889 |  |  |  |  |
| Sample165 | 0.921 |  |  |  |  |
| Sample166 | 0.943 |  |  |  |  |
| Sample167 | 0.903 |  |  |  |  |
| Sample168 | 0.909 |  |  |  |  |
| Sample169 | 0.893 |  |  |  |  |
| Sample170 | 0.921 |  |  |  |  |
| Sample171 | 0.918 |  |  |  |  |
| Sample172 | 0.876 |  |  |  |  |
| Sample173 | 0.941 |  |  |  |  |
| Sample174 | 0.895 |  |  |  |  |
| Sample175 | 0.898 |  |  |  |  |
| Sample176 | 0.909 |  |  |  |  |
| Sample177 | 0.889 |  |  |  |  |
| Sample178 | 0.917 |  |  |  |  |
| Sample179 | 0.927 |  |  |  |  |
| Sample180 | 0.909 |  |  |  |  |
| Sample181 | 0.920 |  |  |  |  |
| Sample182 | 0.903 |  |  |  |  |
| Sample183 | 0.926 |  |  |  |  |
| Sample184 | 0.922 |  |  |  |  |
| Sample185 | 0.911 |  |  |  |  |

**Table S2. Comparison of major nutrients’ composition between the control and LCD groups.**

|  | **Control group (*n*=50)** | | | | **LCD group (*n*=47)** | | | | ***P^a^*** | ***P^b^*** | ***P^c^*** |
| --- | --- | --- | --- | --- | --- | --- | --- | --- | --- | --- | --- |
|  | **Baseline** | | **Follow-up** | | **Baseline** | | **Follow-up** | |  |  |  |
| Total calorie intake (kcal/d)*^∮^* | 2157.6 | ±35.9 | 2158.0 | ±37.2 | 2174.3 | ±40.1 | 1876.5 | ±40.3*^***^* | 0.778 | <0.001 |  |
| Change |  | 0.39 | ±6.99 |  |  | -297.8 | ±6.12 |  |  |  | <0.001 |
| Carbohydrate (g)*^∮^* | 333.4 | ±5.69 | 332.4 | ±5.59 | 335.1 | ±6.32 | 280.0 | ±5.94*^***^* | 0.863 | <0.001 |  |
| Change | -1.04 | | ±1.25 | | -55.2 | | ±1.36 | |  |  | <0.001 |
| Protein (g)*^∮^* | 85.3 | ±1.42 | 85.4 | ±1.49 | 86.3 | ±1.60 | 79.4 | ±1.76*^***^* | 0.656 | 0.006 |  |
| Change | 0.09 | | ±0.45 | | -6.87 | | ±0.51 | |  |  | <0.001 |
| Fat (g)*^∮^* | 53.9 | ±0.96 | 54.6 | ±1.13 | 54.9 | ±1.07 | 49.4 | ±1.19*^***^* | 0.485 | 0.001 |  |
| Change | 0.73 | | ±0.53 | | -5.50 | | ±0.54 | |  |  | <0.001 |
| Carbohydrate (%)*^∮^* | 61.8 | ±0.12 | 61.6 | ±0.11 | 61.6 | ±0.12 | 59.7 | ±0.12*^***^* | 0.365 | <0.001 |  |
| Change |  | -0.16 | ±0.16 |  |  | -1.95 | ±0.19 |  |  |  | <0.001 |
| Protein (%)*^∮^* | 15.8 | ±0.05 | 15.8 | ±0.05 | 15.9 | ±0.06 | 16.9 | ±0.05*^***^* | 0.397 | <0.001 |  |
| Change |  | 0.01 | ±0.07 |  |  | 1.04 | ±0.08 |  |  |  | <0.001 |
| Fat (%)*^∮^* | 22.5 | ±0.15 | 22.7 | ±0.14 | 22.7 | ±0.17 | 23.7 | ±0.15*^***^* | 0.265 | <0.001 |  |
| Change |  | 0.26 | ±0.19 |  |  | 0.93 | ±0.21 |  |  |  | 0.020 |

Mean ± SE.^∮^tested following logarithmic transformation. *P^a^*-values derived from an independent *t*-test at baseline between groups. *P^b^*-values derived from an independent *t*-test at follow-up between groups. *P^c^*-values derived from an independent *t*-test at changed value between groups. *^*^p*<0.05, *^**^p*<0.01, *^***^p*<0.001 derived from a paired *t*-test to compare baseline and follow-up within each group.
